# Validation of the Spanish Blood Donation Knowledge Questionnaire (BDKQ-Spain) Through University–School Partnerships

**DOI:** 10.3390/healthcare14111568

**Published:** 2026-06-03

**Authors:** Laura Alonso Martínez, Diego Serrano Gómez, Laura Sánchez Gómez, Ángela Pascual Ocejo, Lucía Toimil Septién, Rubén Santamaría San Martín, Edson Zangiacomi Martinez, Miriane Lucindo Zucoloto

**Affiliations:** 1 Department of Health Science, Faculty of Health Science, Universidad de Burgos, 09001 Burgos, Spain; 2 High School Félix Rodríguez de la Fuente, 09007 Burgos, Spain; 3 Department of Social Medicine, Ribeirão Preto Medical School, University of São Paulo, Brazil—FMRP-USP, Av, Bandeirantes, 3.900, Ribeirao Preto 14049-900, Brazil

**Keywords:** blood donation, healthcare, health education, interinstitutional relations, validation

## Abstract

**Background/Objectives:** Blood donation knowledge is increasingly recognised as a relevant construct in health education research due to its role in supporting voluntary donation and informing evidence-based strategies that promote preventive behaviours and civic engagement. This study aimed to develop and validate the Spanish version of the Blood Donation Knowledge Questionnaire (BDKQ22-Spain) through university–school partnerships. The BDKQ22-Spain consists of 22 multiple-choice items, each with a single correct answer, designed to assess knowledge about blood donation. **Methods:** Psychometric evaluation was conducted within a quasi-experimental study involving 228 responses from individuals aged 17 to 51 years (M = 24.12, SD = 7.75), comprising high school and university students enrolled in health and education programmes. **Results:** Factor analysis supported a two-factor structure for the BDKQ22-Spain, confirming its construct validity and reliability (KR-20 = 0.85) for measuring blood donation knowledge in Spanish. The results identified higher levels of knowledge among women, nursing students, and individuals with previous blood donation experience, while lower scores were observed among minors, future teachers, and therapy students. However, both minors and future teachers showed significant improvements following the intervention, reaching levels comparable to those of nursing students. These findings highlight specific knowledge gaps across sociodemographic and academic groups. **Conclusions:** The BDKQ22-Spain represents a valid and reliable tool for assessing blood donation knowledge, supporting its use in health and education settings to guide future research and targeted educational strategies.

## 1. Introduction

Blood donation is a benevolent and voluntary act that sustains healthcare systems worldwide by saving lives through transfusions [1,2]. Despite its critical role, shortages of safe and adequate blood persist in many countries [3]. From 2008 to 2018, voluntary unpaid blood donations worldwide rose by 10.7 million units; however, fewer than 1% of the global population donates on a regular basis [4]. The WHO recommends that at least 3% of the population should donate blood to ensure both safety (through haemovigilance programmes) and an adequate supply by blood group within health systems [4,5]. Inadequate availability directly affects patients with chronic conditions, surgical needs, and those with emergencies, placing health systems under strain [6].

Donation rates are strongly influenced by national wealth and health infrastructures. In high-income countries, the rate stands at 31.5 donations per 1000 people, compared with only 5 in low-income countries [7]. Income levels also affect plasma medicine production [8], underscoring the need for policies that ensure equitable blood access across all socioeconomic contexts [5,9]. Countries differ in their approaches, spanning legal and non-legal policy and practice, for instance, to addressing matters pertaining to blood donation [9,10,11]. The decision to donate blood is strongly influenced by public awareness about donation, especially in countries where donation is voluntary [12,13]. Conversely, a lack of knowledge about eligibility criteria, the blood donation process, and widespread misconceptions contributes to the global dearth of voluntary donors [4,13]. Individuals with limited knowledge about blood donation often assume they are ineligible to donate, which reinforces this misconception and reduces donor rates [13]. Recognising these factors underscores the importance of developing awareness interventions and globally reliable measurement scales to assess knowledge of blood donation, as increased knowledge and, consequently, greater awareness is closely associated with higher donation rates [14,15]. It is therefore essential to design instruments that are appropriately tailored to this issue.

## 2. Literature Review

### 2.1. Blood Donation Knowledge Questionnaire (BDKQ)

The incidence of blood transfusions in Brazil is rising by around 6% each year, while the proportion of voluntary blood donors has stagnated at roughly 1.8% of the population, well below of the WHO’s recommended target of 3%. The multifaceted causes of donor shortages in low- and middle-income countries, particularly Brazil, remain poorly understood despite ongoing efforts to investigate the perceptions, motivations, and barriers surrounding voluntary blood donation [10,11]. One such initiative is the Blood Donation Knowledge Questionnaire (BDKQ-Brazil), designed to measure understanding and awareness related to blood donation [14].

While its application in Brazil has provided valuable insights, showing, for example, that women, donors, and health professionals generally achieved higher levels of knowledge than other groups [14,15], the instrument has not yet undergone full psychometric validation, including exploratory factor analysis (EFA) and confirmatory factor analysis (CFA) [15]. Furthermore, no validated equivalent exists in Spanish, as existing measures assess attitudes towards blood donation rather than knowledge [16,17,18]. In this context, adapting and validating the BDKQ for Spanish-speaking populations represents a critical step towards strengthening educational strategies and awareness campaigns in different cultural settings [16,18]. Such validation is not a mere translation exercise, but rather a process that must consider cultural, historical, political, economic, legislative, educational, and personal experiences factors to guarantee its adequacy and validity in Spanish-speaking contexts [16,19]. Cultural nuances can influence how scale items are interpreted, reflecting national differences in knowledge, values, traditions, and practices surrounding blood donation [20]. By addressing these nuances, the BDKQ-Spain can contribute meaningfully to research, intervention, and policy development aimed at improving voluntary blood donation rates.

### 2.2. Blood Donation in Spain: Knowledge, Awareness, and Challenges

In Spain, legislation stipulates that the donation of blood and blood components must be a voluntary and benevolent act, undertaken by individuals of legal age [21,22,23]. According to the Spanish Government [24], 2.3% of Spaniards donated blood, which is below the WHO target of over 3% [4]. Nevertheless, Spain surpasses the global average, with 38 donors per million, showing a marked difference compared with neighbouring countries such as Portugal and France, which report 24.8 and 23.2 donors per million, respectively [17,25]. Therefore, in Spain, the interplay between public policies, initiatives, and awareness campaigns aimed at enhancing knowledge is crucial in positively influencing participation in blood donation [18].

Previous studies highlight that health education is an essential tool for enhancing social welfare and reducing inequalities, playing a pivotal role in the education of young people and the prevention of health problems [4,16,26]. Furthermore, awareness of the donation process and eligibility criteria increases the likelihood of participation, whereas misconceptions can act as barriers [13,27]. The perception of being medically unfit frequently deters potential donors unnecessarily [17].

Predispositions towards blood donation are generally positive in Spain; however, several concerns and barriers to participation have been identified [16]. These include fear of needles or the blood collection process, limited information about the procedure, low awareness, perceived side effects or associated risks, lack of time or availability, and insufficient motivation to donate [3,16]. Such barriers may vary across individuals and contexts and are important to address in order to encourage blood donation [28]. While these obstacles have been widely documented, there is limited understanding in Spain of how they present in younger populations or among individuals training to become educators [18].

### 2.3. The Role of Young People and Future Teachers: Implementing Active Methodologies in Educational Interventions

Blood donation is a critical public health activity, and young people and future teachers represent two groups that are key in its promotion in Spain [16,17,18]. Minors are a significant group of potential donors, and research indicates that early education on blood donation fosters greater long-term commitment [8]. Similarly, future teachers hold the potential to influence blood donation awareness in children and adolescents, shaping attitudes and behaviours from an early age [17,29]. Nevertheless, studies report considerable variation in knowledge and attitudes about blood donation among these groups [14,16,30], highlighting the need for targeted interventions.

To address this need, structured training and awareness programmes for future teachers are necessary to equip them with the skills required to promote blood donation effectively within their future classrooms [30]. In addition, partnerships between educational institutions and blood donation organisations are also critical, supporting school-based initiatives and facilitating experiential learning [17]. Empowering teachers and students with both knowledge and practical skills promotes blood donation as a life-saving and benevolent act [18]. This approach also aligns with the Sustainable Development Goals, contributing to the development of responsible, health-literate citizens [31].

The integration of active methodologies in education is essential to increase knowledge about blood donation among students [16]. Approaches such as project-based learning (PBL) and service have been shown to increase prosocial behaviours, including the willingness to donate blood [16,32,33]. Institutional support through partnerships with non-profit organisations allows educators and students to address disparities in donation and actively contribute to improving learning (SL), thus fostering collaboration, digital literacy, scientific skills, and effective problem-solving, all in the context of blood supply [30].

### 2.4. Research Gap

Knowledge about blood donation is a key factor in increasing the number of donors, as understanding donation processes, procedures, and facts enables informed decisions and fosters sustained commitment [30,34,35]. Despite this, there is a notable lack of validated Spanish instruments specifically assessing blood donation knowledge [17,18,30]. Existing scales primarily focus on attitudes, fears, and willingness to donate, overlooking the critical role of knowledge in raising awareness and promoting active participation [8,16,36,37,38].

This gap underscores the need to develop and validate tools that measure knowledge to support educational interventions and enhance donor recruitment among teachers and young people. Young people and future teachers play a central role in cultivating a society of informed, committed donors [16,17,18,30]. Empirical evidence indicates that early training can strengthen long-term donation behaviours, while teachers can act as multipliers by shaping awareness, attitudes and knowledges from an early age [8,29]. Active project- and service-based learning methodologies offer practical approaches to foster understanding, involvement, and behavioural change [16,33]. However, few studies have explored active interventions targeting these groups, and there is limited evidence of the effectiveness of programmes designed to increase knowledge about blood donation [14,15,28,30]. Addressing these research gaps is essential to develop and implement effective strategies that equip young people and educators with the knowledge needed to promote blood donation, thereby contributing to increased donor participation in society [18,29]. In addition, this study is based on a transnational collaboration between Brazilian and Spanish researchers. The questionnaire was originally developed by the Brazilian team, and the present study aims to validate it in Spain. This partnership is particularly meaningful because both countries have culturally distinct blood donation systems, making Spain an ideal context in which to examine the instrument’s transferability. The collaboration ensures methodological rigour, appropriate cultural adaptation, and fidelity to the original design, contributing to a broader global understanding of blood donation behaviours.

### 2.5. Objective

This study aims to develop and validate the Blood Donation Knowledge Questionnaire in Spanish (BDKQ-Spain) to provide a reliable measure of blood donation knowledge. It also seeks to evaluate the effectiveness of an active educational intervention, collaboratively designed by Brazilian and Spanish experts, Spanish cross-national and local associations, the university, the high school, and the Brotherhood of Blood Donors, in improving blood donation knowledge. The intervention is expected to enable high school students and future teachers to attain knowledge levels comparable to those of health students, fostering a culture of community engagement and social responsibility. The study also aims to identify baseline differences in knowledge by sex, age, educational course and prior donation experience in line with findings from previous research on blood donation knowledge.

## 3. Methods

### 3.1. Research Design

A quasi-experimental study was conducted to evaluate the educational intervention aimed at increasing blood donation knowledge. In this design, the intervention served as the independent variable, while the improvement in blood donation knowledge was the dependent variable. Concurrently, using the collected data, the BDKQ-Spain scale was validated, and knowledge differences were analysed based on sociodemographic variables, including gender, age, previous donation experience, study course and selection of a Master’s degree in teaching.

### 3.2. Procedure and Intervention

Approval was obtained from the Ethics Committee of the University of Burgos (approval number: IR13/2022). Prior to implementation, permission was first obtained from the school and university administration. Subsequently, the teachers then obtained consent from parents or legal guardians, and consent was also obtained from the students themselves, ensuring three levels of authorization. In the case of adult students, their individual informed consent was required. All procedures complied with institutional and national ethical standards, ensuring participant confidentiality and data protection throughout the study. The intervention was developed following PBL and SL methodologies and implemented in the research project subject in the sixth form over 80 h of tutoring. In response to the needs identified by the participating educational centres in Burgos (Spain), students, teachers, and university tutors collaborated with the Blood Donor Association and Brazilian experts to promote awareness of blood donation.

The educational programme used in this intervention was designed following the guidelines of the Spanish Brotherhood of Blood Donors (blood drops that save lives), the Brazilian and Spanish Government’s Donor Management Manual and the WHO. The contents of the intervention focused on highlighting the relevance of blood donation as a lifesaver, backed up by data on the need for donors. Requirements, including age, weight and health, were reported, and the procedure, quantity, time involved, and safety measures employed were detailed. Advantages for recipients and donors were discussed, as well as possible side effects, and advice was given on how to encourage donation in close circles and organise school campaigns, emphasising the responsibility of donating blood when of age. At the end, a gamification activity created by the researchers entitled *“Donation Case Cards according to Personal Background”* was developed, where groups of 4 participants chose suitable donors for fictitious patients.

The procedure followed during the intervention consisted of several stages. Firstly, all experimental groups completed the questionnaire (pre-test) to assess prior knowledge, and then the intervention was carried out with the individuals in the Master’s degree in teaching and high school groups. The intervention included presentations and gamification activities and lasted three sessions of 2 h each. At the end, the questionnaire (post-test) was applied to the experimental and control groups. The questionnaires were answered in the classes of the participating teachers and lecturers, through links sent to the institutional mail or posted on the platform of their classes. In addition, the teachers and lecturers communicated the results to the students during their classes in order to increase knowledge about blood donation.

### 3.3. Instrument

A questionnaire was created via Microsoft Forms, containing informed consent forms and questions with personal and sociodemographic data, as well as a standardised scale (BDKQ24-Spain) and open-ended questions for additional comments, and it was mailed to participants and legal guardians.

The BDKQ-Brazil [14] assesses knowledge about blood donation through 24 items with three, four or five response options that address the requirements, process, and frequently asked questions based on Brazilian blood donation regulations [14,15]. The calculation of the scores consists of summation of the items after having assigned a value of 1 for correct answers and 0 for incorrect answers. The cut-off points established for knowledge about donation were 0–13 (low), 13–19 (medium) and 19–24 (high). The scores between the brackets are not exactly equal due to variation in the difficulty of the questions in the questionnaire. Higher scores on the BDKQ-Brazil indicate greater knowledge of blood donation [15]. In this study, a mean BDKQ24-Spain score of 14.88 (*N* = 228, *Min* = 2, *Max* = 24 and *SD* = 5.26) was obtained.

To establish content validity, the BDKQ24-Spain was adapted to Spanish through the translation and retranslation of the BDKQ-Brazil validated in Portuguese following the guidelines of the University of Michigan [39]. This process included a double translation carried out by experts in the field, as well as a piloting phase to ensure its adequacy and comprehension in the Spanish context. The questionnaire was further reviewed by Brotherhood of Blood Donors associations and by five experts in blood donation and education, including three from Spain and two from Brazil, as well as through group sessions with high school and university students to evaluate comprehension and usability. As a result of this process, several modifications were made. Specifically, in items 5, 8, 10, and 16, the term “Brazil” was replaced with “Spain”. In addition, in items 3, 12 and 14, the Spanish Health System was added, and in item 20, millilitres were added to clarify the measure of capacity of a cup to facilitate the answers. Therefore, following the validation process, the BDKQ-24 was established through content validity, and the final BDKQ-22 Spain was obtained from the factorial analysis, and both are provided in the Supplementary Materials, which also include guidance on their use and interpretation. The cut-off points of the BDKQ-22 Spain for knowledge about donation were low knowledge of blood donation for scores of 10 or below; moderate knowledge for scores between 11 and 17; and high knowledge for scores of 18 or above. 

### 3.4. Participants

**Figure 1:**
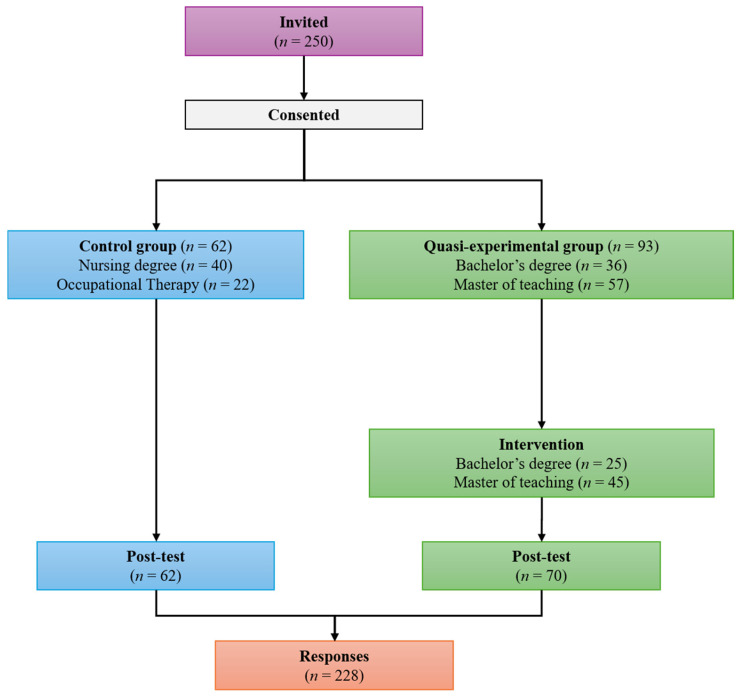
Flow diagram of participant selection and testing process.

The study sample consisted of 228 responses from individuals aged 17 to 51 years (*M* = 24.12, *SD* = 7.75), of whom 156 identified as female and 72 as male (see Table 1). The control group consisted of 62 students, including 40 from the Nursing Degree programme (*M* = 23.08, *SD* = 7.02) and 22 from the Occupational Therapy Degree programme (*M* = 22.29, *SD* = 4.66), of whom 45.16% were donors. The quasi-experimental group consisted of 166 responses, including 61 from students pursuing a Bachelor’s degree (*M* = 17.05, *SD* = 0.20) and 105 from students pursuing a Master’s degree in teaching (*M* = 29, *SD* = 7.31), of which 36.14% were donors. Regarding the quasi-experimental group, 36 participants from the Bachelor’s degree group and 57 from the Master’s degree in teaching group completed the pre-test prior to the intervention. However, during the interventions, 11 participants from the Bachelor’s degree group and 12 from the Master’s degree in teaching group did not attend. Consequently, only 25 Bachelor’s students and 45 students undergoing teacher training completed both the pre-test and post-test and took part in the intervention. In addition, three students from the Master’s degree in teaching group joined the interventions, but only completed the post-test. Furthermore, Figure 1 was created to illustrate the flow of participant selection and the testing process. To evaluate potential attrition bias, we examined whether missingness at post-test was associated with baseline characteristics. First, gender distribution was compared by course and pre-test/post-test status. Chi-square tests showed no significant differences in attrition by gender in either the high school group (*χ*^2^ = 36, *p* = 0.42) or the Master of teaching group (*χ*^2^ = 45.24, *p* = 0.46). Age differences were assessed by course level at pre-test and post-test. Independent-samples *t-tests* indicated no significant differences in age between completers and non-completers in the high school group (*t* = 0.27, *p* = 0.40) or the Master of teaching group (*t* = 0.85, *p* = 0.40). Finally, pre-test performance was compared between completers and non-completers within each course. ANOVA results showed no significant differences pre-test scores between completers and non-completers in either the high school group (*F* = 0.33, *p* = 0.90) or the Master of teaching group (*F* = 0.30, *p* = 0.99). Overall, these findings indicate that attrition was likely missing at random and is unlikely to bias the study’s main comparisons.

A total of 228 responses to the questionnaire were obtained from the experimental and control groups. Inclusion criteria were established based on the selected educational levels, and participants who did not sign the informed consent form were excluded. See Table 1 for more sociodemographic data analysed in the study.

### 3.5. Data Analysis

The BDKQ-Spain was validated, and its factor structure was confirmed through both EFA and CFA (construct validity). This analysis was conducted using the JASP 0.16.4 X64 software. A promax oblique rotation was applied, and estimation was carried out through the Maximum Likelihood method [40]. For indices constructed from dichotomous (yes/no) scales, polychoric/tetrachoric correlations were employed to ensure appropriate validation. For dichotomous items (e.g., yes/no responses), reliability is best assessed using the Kuder–Richardson (KR) formula, namely KR-20 when item difficulty varies and KR-21 when items are assumed to have equal difficulty. A KR value above 0.70 indicates good reliability, as the formula is specifically designed for binary data and offers a more accurate estimate of internal consistency than Cronbach’s alpha [41].

It is important to note the adequacy of the sample size for this analysis, since the number of responses was 10 times greater than the number of items [40]. Internal consistency was assessed through correlations, covariances, Cronbach’s Alpha (*α*) of the deleted items and Omega de McDonald’s (*ω*) of the deleted items. External and criterion validity were examined by relating sociodemographic variables to questionnaire scores [42]. The internal structure was first examined using EFA to explore the underlying dimensions, and the results were subsequently confirmed through CFA [40]. We established 0.30 as the minimum factor loading cutoff and selected items with higher loadings. These analyses are adequate to establish differences in the structure of the BDKQ and the identification of underlying scales. According to Putnick and Bornstein’s [43] criteria, invariance is considered adequate if the differences in ΔCFI are ≤0.01, differences in ΔRMSEA are ≤0.015, and those in ΔSRMR are ≤0.015. The associations with background variables of the BDKQ-Spain will be determined by comparing them with the sociodemographic variables [40]. These analyses are suitable for establishing differences in the structure of the BDKQ-Spain and the identification of underlying scales.

In the study, descriptive and inferential analyses were conducted using IBM SPSS-27 to evaluate the intervention and further support construct validation. In order to address the main objective, Student’s *t-tests* and *ANOVA* followed by Bonferroni’s post hoc test were used to compare independent samples. In addition, Pearson’s correlation was used to analyse relationships between BDKQ-Spain scores and age. A significance level of *p* < 0.05 was established [42], and effect size was calculated using Cohen’s *d* statistic, considering *d* = 0.20 (small), *d* = 0.50 (medium) and *d* = 0.80 (large). To calculate the effect size of the ANOVA test, the partial eta squared statistic (*η_p_*^2^) was used, with *η_p_*^2^ = 0.01 (small), *η_p_*^2^ = 0.06 (medium) and *η_p_*^2^ = 0.14 (large). Correlations are considered small/medium/strong with *r* values of 0.10/0.30/0.50, respectively.

## 4. Results

### 4.1. Reliability Analysis of BDKQ24-Spain

Table 2 shows the internal consistency of the BDKQ24-Spain, the means, the variances of its 24 items, the item–total correlation coefficients and the *αs* of the deleted item. The correlations between each item and the corrected scale score were mostly above 0.30, except for items 12, 14 and 22. In addition, item 22 was not significantly correlated with the total score. However, following the recommendations of Zyl and Klooster [44] in the item selection process, items that differentiate extreme groups and whose *α* values are appropriate should not be removed, indicating that it was not necessary to remove any of these items [40]. In this study, the BDKQ24-Spain demonstrated good reliability, with a KR-20 coefficient of 0.85 and a KR-21 coefficient of 0.83. The mean questionnaire score was 14.88 (*N* = 228, *Min* = 2, *Max* = 24, *SD* = 5.26, σ^2^ = 27.65). Finally, the BDKQ24-Spain also obtained high reliability (*α* = 0.87 and ω = 0.86) and a small inter-item correlation of 0.22.

Table 3 presents the difficulty and discrimination indices of the BDKQ24-Spain items in the study sample. Most items fell within the acceptable range for both difficulty and discrimination, which is considered optimal. However, items 3 and 12 were classified as easy and non-discriminatory, as they did not exceed the threshold of 0.26 [41]. It is therefore recommended that these items be removed from further analysis. Accordingly, they will not be included in subsequent factor analyses to evaluate their structural relevance.

### 4.2. EFA of BDKQ22-Spain

After analysing these data, the appropriateness of conducting a factor analysis [40] was assessed and showed an adequate Kaiser, Meyer and Olkin test (KMO) value of 0.86 and a significant Bartlett’s test of sphericity (*χ*^2^ = 17155.40, *gl* = 231, *p* < 0.001), indicating significant relationships between items and the appropriateness of conducting these factor analyses.

An EFA was performed by applying a promax oblique rotation, based on polychoric/tetrachoric correlations, and Maximum Likelihood estimation was used to maximise the probability of observing the given data [45]. Following the EFA, two factors were identified in the factorial solution (see Table 4). The first factor explains 26.4% of the total variance and the second factor explains 21.8%.

The parallel analysis suggested that the two factors should be retained. All the items load above 0.40. The results of this study suggest that this structure should be tested through a CFA, comparing both one-factor and two-factor models.

### 4.3. CFA of BDKQ22-Spain

This analysis confirmed the results previously identified in the EFA [40,45]. Using a Maximum Likelihood approach in JASP, we obtained standardised estimates, direct and indirect effects, and covariance values [41]. A confirmatory factor analysis is considered to show good model fit when the CFI, NNFI, RNI, and TLI exceed the recommended threshold of 0.90; the SRMR and RMSEA fall below 0.08; and the CMIN/DF value is less than the accepted cut-off of 3 [40,41]. Table 5 displays the CFA indices for two models of the BDKQ-22. The one-factor model exhibited inferior fit compared to the two-factor model.

In the original version, it was theoretically considered appropriate to keep the questionnaire unifactorial. However, in this study, the two-factor model was deemed more suitable than the one-factor model, although the modest CFA indices indicate the need for further analysis. In this study, the Subscale-1 (Blood Donation Criteria and Conditions) of the BDKQ22-Spain demonstrated high reliability, with a KR-20 coefficient of 0.80, a KR-21 coefficient of 0.76, an *α* value of 0.80 and *ω* = 0.80. The mean score was 5.86 (*N* = 228, *Min* = 0, *Max* = 10, *SD* = 2.77, *σ*^2^ = 7.66). The Subscale-2 (Blood Donation Process and Regulations) of the BDKQ22-Spain also demonstrated high reliability, with a KR-20 coefficient of 0.82, a KR-21 coefficient of 0.76, an *α* value of 0.79 and *ω* = 0.80. The mean score was 7.17 (*N* = 228, *Min* = 0, *Max* = 12, *SD* = 3.10, *σ*^2^ = 9.56). Overall, the BDKQ22-Spain demonstrated high reliability, with a KR-20 coefficient of 0.85, a KR-21 coefficient of 0.84, an *α* value of 0.86 and *ω* = 0.85. The mean questionnaire score was 13.03 (*N* = 228, *Min* = 0, *Max* = 22, *SD* = 5.14, *σ*^2^ = 26.38).

### 4.4. Invariance of the BDKQ-22 Across Gender

In this case, measurement invariance refers to the consistency of the BDKQ22-Spain measurements across gender. Initially, the configural invariance model was tested, revealing a unifactorial structure between groups with satisfactory fit indices (*CFI* = 0.75; *RMSEA* = 0.08; *SRMR* = 0.08). Next, the metric invariance model was examined, indicating adequate fit (*CFI* = 0.75; *RMSEA* = 0.08; *SRMR* = 0.09) with minimal differences from the configural model. Scalar invariance, which incorporates equal intercepts, showed a good fit (*CFI* = 0.69, *RMSEA* = 0.09, *SRMR* = 0.10), although there were unexpected changes in the CFI compared to metric invariance because the ΔCFI was >0.01. The strict invariance model, which incorporates restricted error variances, fit well (*CFI* = 0.69, *RMSEA* = 0.09, *SRMR* = 0.10). Comparisons between models were based on established criteria [43]. Given the moderate fit of the baseline models, the evidence for configural and scalar invariance should be interpreted with caution. Rather than claiming full invariance, we present these BDKQ-22 findings as exploratory, indicating only approximate equivalence between men and women.

### 4.5. Analysis of the BDKQ22-Spain Scores in Relation to Intervention and Sociodemographic Variables

The performance of the BDKQ22-Spain was evaluated in terms of hits and misses. Questions with low hit rates related to donation barriers and myths, such as question 13 (blood expiry) with a 53.10% hit rate, and questions 8 (menstruation), 9 (age of donation), 14 (acquiring diseases) and 20 (volume of blood donated) with less than a 50% hit rate. In addition, questions 10 (lactation), 11 (volume donated per donation), 15 (period between donations) and 24 (clotted or diluted blood) scored less than 40% correct answers. The rest of the questions had hit rates between 60% and 89%. The implemented intervention had a positive impact on increasing knowledge about donation, as reflected in the scores obtained in the BDKQ22-Spain and in the comments made by the participants in the sessions. A linear mixed-effects model was used to examine the intervention influence of course (high school, Master’s in teacher education, Occupational Therapy and nursing) and time (pre-test versus post-test) on knowledge about blood donation (BDKQ-22). The analysis revealed a significant main effect of course (*F*(1, 228) = 27.48, *p* < 0.001), with students in nursing and Master’s programmes scoring higher overall than those in high school and Occupational Therapy. There was also a significant main effect of time (*F*(1, 228) = 223.23, *p* < 0.001), showing that knowledge scores improved from pre-test to post-test across all groups. The interaction of course and time was not significant (*F*(1, 228) = 2.82, *p* = 0.09), suggesting that the extent of improvement over time was similar across courses.

Statistically significant differences were also observed between the average scores of the pre-test and post-test, as well as between the control and quasi-experimental groups. Factors such as previous donation experience, knowledge of blood group, higher educational level and older age were correlated with greater knowledge in this area. Science and health science students initially showed greater knowledge than their peers in other disciplines. A slight positive correlation (*r* = 0.19, *p* < 0.003) between the BDKQ22-Spain score and age stands out, indicating that the older the age, the more correct answers are obtained. These results are explored in detail in Table 6 and Table 7, which present the differences in the BDKQ22-Spain scores with respect to the criterion variables assessed.

## 5. Discussion

This study successfully achieved its objectives by developing and validating the Blood Donation Knowledge Questionnaire in Spanish (BDKQ-Spain), which exhibited strong reliability and validity. In addition, the educational intervention effectively increased knowledge about blood donation among high school students and future teachers. After the intervention, participants’ knowledge levels appeared broadly comparable to those of nursing students, indicating that active, contextually grounded educational programmes may help reduce knowledge gaps across different educational groups.

The BDKQ22-Spain, adapted from the BDKQ-Brazil, performed well psychometrically [14]. In adapting the questionnaire, two items from the BDKQ-Brazil were removed because they were too easy and reflected principles already well known in Spain’s public health system. These items were the only ones referencing Spain, so their removal aids broader applicability in other Spanish-speaking countries. Their exclusion also helped avoid ceiling effects and improved the tool’s ability to discriminate between different levels of knowledge [41]. The EFA and CFA results aligned more closely with a two-factor model than with a unifactorial structure. These findings cannot be directly compared with those of the original study by Zucoloto and Martínez [14], as they did not carry out factor analyses, although they did theoretically suggest that the scale functions best as a unidimensional measure. Given the modest indices reported, the existence of distinct subscales still requires further confirmation, and additional analyses are therefore recommended [45].

Our findings indicate that previous experience of blood donation was a strong predictor of higher knowledge, with 36.70% of participants having donated blood. This reflects a positive attitude towards benevolent behaviour, consistent with findings from other Spanish studies [16,17,18]. This benevolent mentality may have been reinforced by donation campaigns within the university environment, organised in collaboration with local associations such as the Brotherhood of Blood Donors, which also took part in this study. These campaigns enhance knowledge about blood donation, which primarily motivated participants to support the blood bank and help maintain its reserves [3,6,17,18].

Nonetheless, several areas of limited knowledge linked to barriers to blood donation were also identified. These included fears of disease transmission, fear of needles and blood, concerns about possible negative health effects, as well as discomfort and weight-related issues, consistent with findings reported by Haw et al. [3] and Chand et al. [6]. The persistence of these concerns within our sample is particularly striking given the high level of awareness of blood donation safety measures (with over 90% answering correctly). More concerning still is that fewer than half of the participants correctly recognised that, under haemovigilance protocols, disease transmission through blood donation is unlikely within the Spanish health system, reflecting the knowledge barriers described in other studies [6,30].

When compared to prior research, blood donation knowledge in our quasi-experimental pre-test group was lower that reported in the original version validated by Zucoloto and Martinez [14] among Brazilian health centre users, whose mean score was 15.35 (SD = 3.25, N = 1055). In our study, however, knowledge levels improved after the intervention, ultimately surpassing those of the Brazilian participants. Similarly, when compared with the study by Zucoloto et al. [15], conducted among health science students (*M* = 13.67; *SD* = 7.96, *N* = 340, 81.50% female) of a comparable age and sample to ours, our pre-test scores across most groups indicated lower levels of blood donation knowledge. The post-test results, however, showed higher levels of knowledge than those reported by Zucoloto et al. [15], with Occupational Therapy students in our study achieving mean scores comparable to those in their research. This is consistent with previous research [17,18], which suggests that nursing students have greater knowledge due to their involvement in different stages of the blood donation process. The research by Zucoloto et al. [15] also indicated that health science students possessed the strongest background knowledge overall. However, it also showed that students of Occupational Therapy, nutrition, physiotherapy, and speech therapy had less knowledge than their peers in medicine and nursing, reflecting differences in the content covered within their respective disciplines.

As in the studies by Zucoloto and Martínez [14] and Zucoloto et al. [15], our study also identified demographic differences in blood donation knowledge, with women, older participants, and previous donors demonstrating higher levels of knowledge. These differences in knowledge are attributed to several factors, including accumulated experience over time, repeated exposure to the donation process, greater interest and awareness in the role of gender in health, culturally influenced prosocial behaviours, and greater accessibility to information and educational programmes [2,5,6,8]. The relationship between these factors and willingness to donate may vary and be influenced by other sociocultural and individual factors [15]. Nevertheless, in line with our findings and previous research, the most notable factor appears to be the level of education received [2,14].

Therefore, the present intervention has demonstrated that health education, applying active methodologies, is associated with the acquisition of knowledge about blood donation as these methodologies engage students, promoting deeper understanding and long-term learning, consistent with previous studies [26,33]. This positive outcome is also aligned with findings from other studies emphasising the benefits of integrating hands-on experiences, and discussions related to donation, which connect theory with practice, enhance intrinsic motivation to learn, and foster critical reflection [16,30].

Our study, consistent with previous research, underscores the importance of educating adolescents, future teachers, and health professionals about blood donation to foster awareness and a culture of solidarity from an early age [15]. Addressing donation among minors promotes benevolent values, encourages family involvement, and supports the development of a sustainable donor culture [1,18]. The participation of these groups in this research contributes to fostering a caring society that supports blood banks in maintaining an adequate supply of blood for those in need [15,17,28]. Moreover, as highlighted in this study and previous research, equipping health and education professionals with knowledge about blood donation is essential for patient safety, demystifying myths, recognising eligibility criteria, and managing potential complications [17,18].

### Strengths and Limitations

The strengths of the study are that the BDKQ22-Spain questionnaire demonstrated strong psychometric properties supported by reliable analyses, EFA and CFA, thereby confirming its validity and reliability for assessing blood donation knowledge in Spanish-speaking populations. The BDKQ22-Spain provides a standardised tool for future evaluations. The intervention effectively enhanced knowledge about blood donation among high school students and future teachers. By incorporating innovative pedagogical approaches, such as SL and PBL, the study fostered active student engagement and meaningful, context-based learning in both secondary and higher education settings. As this study was conducted in real educational settings, the research enhanced the practical relevance of its findings, while active methodologies supported the acquisition of practical knowledge about blood donation and encouraged informed health-related decision-making. Moreover, the quasi-experimental design, involving 228 responses from diverse educational and health-related backgrounds, provided a broad understanding of the intervention’s impact.

Despite the valuable findings obtained in this study, several limitations should be considered. The validation of the BDKQ-22 Spain did not include comparisons with other validated instruments, and the CFA indices supporting the two-subscale structure were modest. The two items removed during the EFA were specific to Spain, while the remaining items are broadly applicable across Spanish-speaking countries; nevertheless, it is recommended that the questionnaire be tested in other Spanish-speaking countries. The quasi-experimental design and sample diversity limited the ability to draw generalised findings. Furthermore, it is recognised that knowledge alone does not automatically translate into behavioural change, as other unmeasured psychosocial factors (e.g., self-efficacy) also play a critical role. Future studies should carry out factor analysis in additional samples to confirm the structure of the questionnaire. They should also adopt more robust designs, including larger samples, and randomised cluster approaches to evaluate long-term effects and strengthen evidence. Additionally, expanding participant diversity across educational contexts would further enhance generalizability and should incorporate additional behavioural determinants, such as self-efficacy.

## 6. Conclusions and Implications

The objectives of this study have been successfully achieved, confirming the reliability, validity and relevance of the BDKQ22-Spain among high school students as well as educational and healthcare professionals. The educational intervention, conducted in collaboration with Brazilian and Spanish experts and the Brotherhood of Blood Donors, had a positive impact on participants, as evidenced by an increase in their knowledge about blood donation. High school students, prospective teachers, and Occupational Therapy students were found to have lower levels of knowledge compared with nursing students. However, following the intervention, high school students and future teachers achieved knowledge levels comparable to those of nursing students. These findings underscored the effectiveness of active learning methodologies and highlight the importance of health education in enhancing knowledge about blood donation within this population. The study also showed the value of partnerships between universities and schools in enhancing the quality of health education. Educational interventions of this kind are therefore essential for fostering awareness and knowledge of blood donation among high school students and future professionals in both health and education sectors.

## Figures and Tables

**Table 1:** Sociodemographic characteristics of the study population.

Variables	Total
**Gender**	
Woman	156 (68.40%)
Man	72 (31.60%)
**Education**	
Master’s Degree in Teaching	105 (46.10%)
Second Baccalaureate	61 (26.75%)
Nursing Degree	40 (17.50%)
Degree in Occupational Therapy	22 (9.60%)
**Previous experiences related to donation** (donor, health care provider, donor family members, transfused or health care provider and receiving transfusion)	
Donation history	181 (79.40%)
No history	47 (20.60%)
**Blood group**	
A	66 (28.90%)
B	20 (8.80%)
0	48 (21.10%)
AB	9 (3.90%)
I do not know	85 (37.30%)
**Total**	228 (100%)
**Specialty of the Master’s Degree in Teaching**	
History	12 (11.40%)
Language	11 (10.50%)
English	10 (9.50%)
Biology	17 (16.20%)
Physics	15 (14.30%)
Mathematics	16 (15.20%)
Technology	6 (5.70%)
Job training	6 (5.70%)
Educational guidance	10 (9.50%)
Socio-community intervention	2 (1.90%)
**Total**	105 (100%)

**Table 2:** Reliability of the BDKQ24-Spain.

Item	M	DT	M ^1^	Var. ^2^	CI-T ^3^	A ^4^	ω ^5^
BDKQ1	1.36	0.48	47.91	71.07	0.35	0.86	0.86
BDKQ2	2.32	0.80	46.96	66.04	0.58	0.86	0.85
BDKQ3	1.13	0.46	48.14	71.53	0.31	0.86	0.86
BDKQ4	2.13	0.43	47.14	71.38	0.36	0.86	0.85
BDKQ5	2.17	0.53	47.10	69.70	0.48	0.86	0.85
BDKQ6	2.07	0.58	47.20	69.55	0.44	0.86	0.86
BDKQ7	2.23	0.57	47.04	69.25	0.49	0.86	0.85
BDKQ8	1.79	0.80	47.48	67.26	0.48	0.86	0.86
BDKQ9	1.79	0.84	47.48	67.38	0.44	0.86	0.85
BDKQ10	2.04	0.81	47.23	67.60	0.45	0.86	0.85
BDKQ11	1.98	0.81	47.29	66.50	0.53	0.86	0.85
BDKQ12	2.02	0.26	47.25	72.94	0.26	0.87	0.86
BDKQ13	2.11	0.68	47.16	67.97	0.51	0.86	0.85
BDKQ14	1.59	0.66	47.68	70.92	0.25	0.86	0.85
BDKQ15	1.98	0.85	47.29	65.24	0.60	0.86	0.85
BDKQ16	2.17	0.40	47.10	70.77	0.48	0.86	0.85
BDKQ17	1.48	0.81	47.80	66.54	0.53	0.86	0.85
BDKQ18	2.16	0.46	47.12	70.18	0.49	0.86	0.84
BDKQ19	3.00	0.69	46.27	70.359	0.29	0.87	0.85
BDKQ20	3.31	1.250	45.96	62.293	0.52	0.86	0.85
BDKQ21	1.87	1.256	47.40	60.929	0.60	0.86	0.85
BDKQ22	1.81	0.58	47.46	73.884	−0.01	0.87	0.84
BDKQ23	1.39	0.76	47.88	66.201	0.60	0.86	0.85
BDKQ24	3.39	0.71	45.88	68.701	0.43	0.86	0.86

^1^ *M* = scale mean (if item is removed). ^2^ *Var.* = variance of the scale (if the item is removed). ^3^ *IQ-T* = item–total correlation. ^4^ *α* = Cronbach’s Alpha (if item is removed). ^5^ ω = McDonald’s Omega (if item is removed).

**Table 3:** Item difficulty and discrimination indices of BDKQ24-Spain in the sample.

Item	Item Difficulty Index	Interpretation	Item Discrimination Index	Interpretation	Interpretation
BDKQ1	0.64	Average	0.47	Very good	Retain
BDKQ2	0.77	Easy	0.56	Very good	Retain
BDKQ3	0.92	Easy	0.18	Non-discriminatory	Dismiss
BDKQ4	0.80	Easy	0.45	Very good	Retain
BDKQ5	0.69	Average	0.71	Very good	Retain
BDKQ6	0.66	Average	0.39	Good	Retain
BDKQ7	0.63	Average	0.70	Very good	Retain
BDKQ8	0.45	Average	0.29	Good	Retain
BDKQ9	0.47	Average	0.56	Very good	Retain
BDKQ10	0.35	Average	0.56	Very good	Retain
BDKQ11	0.33	Average	0.71	Very good	Retain
BDKQ12	0.93	Easy	0.15	Non-discriminatory	Dismiss
BDKQ13	0.53	Average	0.48	Very good	Retain
BDKQ14	0.40	Average	0.63	Very good	Retain
BDKQ15	0.38	Average	0.52	Very good	Retain
BDKQ16	0.82	Easy	0.52	Very good	Retain
BDKQ17	0.72	Average	0.55	Very good	Retain
BDKQ18	0.76	Easy	0.26	Good	Retain
BDKQ19	0.75	Average	0.51	Very good	Retain
BDKQ20	0.46	Average	0.70	Very good	Retain
BDKQ21	0.62	Average	0.56	Very good	Retain
BDKQ22	0.63	Average	0.87	Very good	Retain
BDKQ23	0.78	Easy	0.61	Very good	Retain
BDKQ24	0.39	Average	0.42	Very good	Retain

**Table 4:** Factor matrix of the final BDKQ22-Spain.

Item	Factors		One Factor
	1	2	
BDKQ1		0.57	0.73
BDKQ2	0.45		0.46
BDKQ4	0.55		0.59
BDKQ5	0.79		0.33
BDKQ6	0.68		0.66
BDKQ7	0.64		0.44
BDKQ8		0.68	0.67
BDKQ9	0.60		0.66
BDKQ10	0.94		0.28
BDKQ11	0.74		0.43
BDKQ13		0.61	0.64
BDKQ14	0.75		0.51
BDKQ15		0.68	0.58
BDKQ16		0.48	0.52
BDKQ17		0.48	0.62
BDKQ18		0.53	0.20
BDKQ19	0.47		0.60
BDKQ20		0.57	0.50
BDKQ21		0.65	0.61
BDKQ22		0.54	0.24
BDKQ23		0.68	0.29
BDKQ24		0.41	0.84

**Table 5:** CFA fit indices in the BDKQ-22.

Model	χ^2^	df	CMIN/DF	CFI	IFI	TLI	RNI	SRMR	RMSEA	Δχ^2^	Δdf
1-Factor	451.56	209	2.16	0.80	0.80	0.78	0.80	0.07	0.07		
2-Factor	355.88	188	1.89	0.86	0.86	0.84	0.86	0.06	0.06	1F − 2F = 95.68 **	21

** *p* ≤ 0.01.

**Table 6:** Differences in BDKQ22-Spain scores in relation to experimental group, research design, gender and previous donation experiences.

Variables		*t*	*df*	*Sig.*	Cohen’s *d*	
					*d*	95% CI
Experimental group, *M* (*SD*)						
Quasi-Experimental Group, 12.67 (5.64), n = 166	Control Group 13.98 (3.26) n = 62	−2.18	226	0.03 *	0.28	−0.55, 0.04
Experimental group post-test, *M* (*SD*)						
Quasi-Experimental Group, 17.23 (2.74), n = 73	Control Group 13.98 (3.32) n = 62	6.21	133	0.001 **	1.07	0.72, 1.44
Experiment pre/post-test design, *M* (*SD*)						
Pre-Test Group, 9.09 (4.67), n = 93	Post-Test Group 17.23 (2.74) n = 73	−13.22	164	0.001 **	2.13	−2.44, −1.69
Previous study pre-test, *M* (*SD*)						
Master’s in Teaching 10.51 (4.94), n = 57	High School 6.83 (3.14) n = 36	3.98	91	0.001 **	0.89	0.41, 1.28
Previous study post-test, *M* (*SD*)						
Master’s in Teaching 17.85 (2.42), n = 48	High School 16.04 (2.94) n = 25	2.65	71	0.01 *	0.67	0.20, 1.18
Gender, *M* (*SD*)						
Woman 13.44 (4.75), n = 156	Man 12.13 (5.82) n = 72	−1.81	226	0.07	0.25	−0.54, 0.02
Experiences with donation, *M* (*SD*)						
Background 13.46 (4.99) n = 181	No Previous History, 11.36 (5.41) n = 47	2.52	226	0.01 *	0.40	0.09, 0.74

* *p* < 0.05 and ** *p* < 0.001.

**Table 7:** Differences in BDKQ22-Spain scores according to study course, Master’s speciality and blood group.

Variables										*F*	*df*	*Sig.*	*η_p_* ^2^
Previous Study, *M* (*SD*)										10.93	3, 228	0.001 **	0.13
Master’s in Teaching ^a^ 13.87 (5.41) ^b^ *n* = 105			High School ^b^ 10.61 (5.48) ^a,d^ *n* = 61			Nursing ^c^ 15.55 (2.05) ^b,d^ *n* = 40		Occupational Therapy ^d^ 11.14 (3.15) ^c^ *n* = 22					
Previous Study Post-Test, M (SD)										34.86	3, 135	0.001 **	0.44
Master’s in Teaching ^a^ 17.85 (2.43) ^b,c,d^ *n* = 48			High School ^b^ 16.04 (2.94) ^a,d^ *n* = 25			Nursing ^c^ 15.55 (2.05) ^a,d^ *n* = 40		Occupational Therapy ^d^ 11.14 (3.15) ^a,b,c^ *n* = 22					
Speciality of the Master’s Degree in Teaching, M (SD)										2.19	9, 105	0.029 *	0.17
History ^a^ 9.58 (6.71) ^d^ *n* = 12	Spanish ^b^ 12.45 (5.47) *n* = 11	English ^c^ 12.7 (5.98) *n* = 10	Biology ^d^ 16.82 (2.63) ^a^ *n* = 17	Physics ^e^ 14.53 (4.41) *n* = 15	Maths ^f^ 13.75 (5.60) *n* = 16	Technology ^g^ 11.83 (6.82) *n* = 6	Vocational training ^g^ 14.33 (5.78) *n* = 6	Counselling ^e^ 16.80 (3.91) *n* = 10	Intervention ^e^ 14 (4.24) *n* = 2				
Blood Group, *M* (*SD*)										9.82	4, 226	0.001 **	0.15
A ^a^ 14.26 (4.31) ^e^ *n* = 66		B ^b^ 14.95 (4.21) ^e^ *n* = 20		O ^c^ 14.21 (4.33) ^e^ *n* = 48		AB ^d^ 16.33 (3.87) ^e^ *n* = 9		Unknown blood group ^e^ 10.43 (5.53) ^a,b,c,d^ *n* = 83					

* *p* < 0.05 and ** *p* < 0.001. Based on the Bonferroni post hoc test (*p* < 0.001), superscript letters were assigned to each group. Groups that carry, in addition to their own superscript letter, one or more letters assigned to other groups differ significantly from those groups.

## Data Availability

The data reported in this manuscript can be approached through contacting the corresponding authors, and it is deposited in a digital repository that can be accessed from the following link: https://doi.org/10.5281/zenodo.12701610.

## Supplementary Materials

The following supporting information can be downloaded at https://www.mdpi.com/article/10.3390/healthcare14111568/s1, Table S1: Blood Donation Knowledge Questionnaire (BDKQ22-Spain).
