# Supplementary material for: Validation of the Spanish Blood Donation Knowledge Questionnaire (BDKQ-Spain) Through University–School Partnerships
Source: Healthcare (Basel). 2026 Jun 3;14(11):1568. doi: 10.3390/healthcare14111568 (PMC13257174; doi:10.3390/healthcare14111568)
Supplement: Supplementary file 1 [file healthcare-14-01568-s001.zip › healthcare-4207398-supplementary.pdf]

**Supplementary material**

**Blood Donation Knowledge Questionnaire (BDKQ-Spain)**

The BDKQ22-Spain comprises 22 items, with the correct responses indicated in bold in Table S1. Each correct response is awarded one point, while incorrect responses receive no score, yielding a total possible range of 0 to 22 points. Items include between two and five response options, of which only one is correct. In this study, the KR20 coefficient of BDKQ-Spain was 0.85 and the $\alpha$ was 0.86, indicating high reliability. The mean questionnaire score was 13.03 ($N$ = 228, $Min$ = 0, $Max$ = 22, $SD$ = 5.14, $\sigma^2$ = 26.38).

The cut-off points established for knowledge about donation were low knowledge of blood donation for scores of 10 or below; moderate knowledge for scores between 11 and 17; and high knowledge for scores of 18 or above.

The average administration time of the questionnaire is approximately 30 minutes. While it can be self-administered, individual administration is recommended. The inclusion of the "I do not know" response option was retained to minimise random guessing and thereby reduce potential sources of bias.

**Table S1.**

*Blood Donation Knowledge Questionnaire (BDKQ22-Spain).*

| Nº | Items | Answers[a] |
|---|---|---|
| 1 | **¿Conoces tu grupo sanguíneo?** (Do you know your blood type?) | 1. **Sí (Yes)**<br>2. No (No) |
| 2 | **Para ser donante de sangre, ¿conoces cuál es el peso mínimo de una persona para poder donar sangre?** (In order to be able to donate blood, what is the minimum weight that a person needs to have?) | 1. 40 kg<br>**2. 50 kg**<br>3. 60 kg<br>4. No lo sé (I do not know) |
| 3[b] | **¿En el Sistema de Salud Español se analiza la sangre donada para comprobar si padece alguna enfermedad que pueda transmitirse a otras personas?** (In Spain, is all donated blood tested in order to verify if it has any disease that can be transmitted to others?) | 1. **Sí** (Yes)<br>2. No (No) |
| 4 | **¿Pueden los menores de 16 años donar sangre?** (Can under 16-years-old individuals donate blood?) | 1. Sí (Yes)<br>**2. No (No)**<br>**3.** No lo sé (I do not know) |
| 5 | **¿Puede una persona embarazada donar sangre?** (Can pregnant women donate blood?) | 1. Sí (Yes)<br>**2. No (No)**<br>3. No lo sé (I do not know) |

| | | |
|---|---|---|
| **6** | **¿Puede una persona que sufre diabetes o presión sanguínea alta donar sangre?** (Can a person who has diabetes or high blood pressure donate blood?) | 1. Sí (Yes)<br>**2. No (No)**<br>3. No lo sé (I do not know) |
| **7** | **¿Puede una persona que tenga o haya tenido algún tipo de cáncer donar sangre?** (Can a person who has or has had any type of cancer donate blood?) | 1. Sí (Yes)<br>**2. No (No)**<br>**3.** No lo sé (I do not know) |
| **8** | **¿Puede una persona que está menstruando donar sangre?** (Can women who are menstruating donate blood?) | **1. Sí (Yes)**<br>2. No (No)<br>3. No lo sé (I do not know) |
| **9** | **¿Hay un máximo de edad para donar sangre?** (Is there a maximum age for blood donation?) | **1. Sí (Yes)**<br>2. No (No)<br>3. No lo sé (I do not know) |
| **10** | **¿Pueden las personas que amamantan a sus hijos donar sangre?** (Can women who are breastfeeding donate blood?) | 1. Sí (Yes)<br>**2. No (No)**<br>**3.** No lo sé (I do not know) |
| **11** | **¿La sangre de un único donador es suficiente para una persona que necesita una transfusión de sangre?** (Is the blood from only one donor enough for one person who needs blood?) | **1. Sí (Yes)**<br>2. No (No)<br>3. No lo sé (I do not know) |
| **12**[b] | **Cuando una persona necesita recibir sangre, ¿tiene que pagar en el Sistema de Salud Español**? (When people need to receive blood, do they have to pay in the Spanish Health System?) | 1. Sí (Yes)<br>**2. No (No)**<br>**3.** No lo sé (I do not know) |
| **13** | **¿La sangre donada debe utilizarse dentro de los 24 días siguientes a la donación, de lo contrario ya no es buena?** (Does donated blood have to be used within 24 h after donation, otherwise it is not good anymore?) | 1. Sí (Yes)<br>**2. No (No)**<br>**3.** No lo sé (I do not know) |
| **14** | **¿Puede una persona contraer una enfermedad donando o recibiendo sangre en el Sistema de Salud Español?** (Currently, can a person acquire a disease by donating blood in the Spanish Health System?) | 1. Sí (Yes)<br>**2. No (No)**<br>**3.** No lo sé (I do not know) |
| **15** | **Es correcto que, si el donante de sangre es varón, puede donar cada 2 meses y la mujer puede donar cada 3 meses.** (It is correct, if the blood donor is male, can he donate every 2 months, and can women donate every 3 months.) | **1. Sí (Yes)**<br>2. No (No)<br>3. No lo sé (I do not know) |
| **16** | **En España, ¿está permitido por ley pagar a una persona para que done sangre?** (In Spain, is it allowed by law to pay a person to donate blood?) | 1. Sí (Yes)<br>**2. No (No)**<br>**3.** No lo sé (I do not know) |

| | | |
|---|---|---|
| **17** | **Cuando alguien dona sangre, ¿la cantidad de sangre en el cuerpo humano vuelve a ser la misma en un plazo de 24-48 horas?** (When someone donates blood, does the amount of blood in the human body return to what it was before within 24–48 h?) | **1. Sí (Yes)**<br>2. No (No)<br>3. No lo sé (I do not know) |
| **18** | **Sí un donante tiene fiebre el día de la donación, ¿puede donar sangre?** (If a donor has a fever on the day of donation, can he donate blood?) | 1. Sí (Yes)<br>**2. No (No)**<br>**3.** No lo sé (I do not know) |
| **19** | **¿Donar sangre te hace ganar o perder peso?** (Does donating blood make you lose or gain weight?) | 1. Perder peso (Lose weigh)<br>2. Ganar peso (Gain weight)<br>3. **Ninguna (Neither)**<br>**4.** No lo sé (I do not know) |
| **20** | **La capacidad de una tacita de café es de 50 ml. Cuando una persona dona sangre, ¿a cuántas tazas de café equivale?** (The capacity of a little coffee cup is 50 mL. When a person donates blood, the equivalent to how many coffee cups are taken?) | 1. 2 – 4 (100-200 ml)<br>2. 5 – 8 (200-400ml)<br>**3. 9 – 10 (450-500ml)**<br>4. 11-20 (550-1000ml)<br>5. No lo sé (I do not know) |
| **21** | **Después de que una persona entre en la sala de donación para donar sangre, ¿cuánto dura el proceso de donación de sangre?** (After a person enters in the donation room to donate blood, how long is the blood donation process?) | **1. 20 min.**<br>2. 40 min a 1 hora (40 m to 1 h)<br>3. Más de 1 hora (More than 1 h)<br>4. No lo sé (I do not know) |
| **22** | **A la hora de donar, ¿debes de ir en ayunas?** (In order to donate blood, should the donor be fasting?) | 1. Sí (Yes)<br>**2. No (No)**<br>**3.** No lo sé (I do not know) |
| **23** | **¿Pueden las personas fumadoras donar sangre?** (Can smokers donate blood?) | **1. Sí (Yes)**<br>2. No (No)<br>3. No lo sé (I do not know) |
| **24** | **¿Puede donarse sangre coagulada o diluida?** (Does donating blood thicken or thin the blood?) | 1. Sí se puede la coagulada (Thin)<br>2. Sí se puede la diluida (Thicken)<br>**3. No se puede ninguna (Neither)**<br>**4.** No lo sé (I do not know) |

[a]The correct answer for each question is highlighted in bold type.

[b]Items removed from the final version of the BDKQ-Spain.
